# Dosimetric Performance and Planning/Delivery Efficiency of a Dual-Layer Stacked and Staggered MLC on Treating Multiple Small Targets: A Planning Study Based on Single-Isocenter Multi-Target Stereotactic Radiosurgery (SRS) to Brain Metastases

**DOI:** 10.3389/fonc.2019.00007

**Published:** 2019-01-22

**Authors:** Taoran Li, Peyton Irmen, Haisong Liu, Wenyin Shi, Michelle Alonso-Basanta, Wei Zou, Boon-Keng Kevin Teo, James M. Metz, Lei Dong

**Affiliations:** ^1^Department of Radiation Oncology, University of Pennsylvania, Philadelphia, PA, United States; ^2^Department of Radiation Oncology, Thomas Jefferson University, Philadelphia, PA, United States

**Keywords:** halcyon, stereotactic radiosurgery (SRS), multi-leaf collimator (MLC), single-isocenter multi-target, truebeam, brain metastase

## Abstract

**Purpose:** To evaluate the dosimetric performance and planning/delivery efficiency of a dual-layer MLC system for treating multiple brain metastases with a single isocenter.

**Materials and Methods:** 10 patients each with 6–10 targets with volumes from 0.11 to 8.57 cc, and prescription doses from 15 to 24 Gy, were retrospectively studied. Halcyon has only coplanar delivery mode. Halcyon V1 MLC modulates only with the lower layer at 1 cm resolution, whereas V2 MLC modulates with both layers at an effective resolution of 0.5 cm. For each patient five plans were compared varying MLC and beam arrangements: the clinical plan using multi-aperture dynamic conformal arc (DCA) and non-coplanar arcs, Halcyon-V1 using coplanar-VMAT, Halcyon-V2 using coplanar-VMAT, HDMLC-0.25 cm using coplanar-VMAT, and HDMLC-0.25 cm using non-coplanar-VMAT. All same-case plans were generated following the same planning protocol and normalization. Conformity index (CI), gradient index (GI), V12Gy, V6Gy, V3Gy, and brain mean dose were compared.

**Results:** All VMAT plans met clinical constraints for critical structures. For targets with diameter < 1 cm, Halcyon plans showed inferior CI among all techniques. For targets with diameter >1 cm, Halcyon VMAT plans had CI similar to non-coplanar VMAT plans, and better than non-coplanar clinical DCA plans. For GI, Halcyon MLC plans performed similarly to coplanar HDMLC plans and inferiorly compared to non-coplanar HDMLC plans. All coplanar VMAT plans (Halcyon MLC and HDMLC) and clinical DCA plans had similar V12Gy, but were inferior compared to non-coplanar VMAT plans. Halcyon plans had slightly reduced V3Gy and mean brain dose compared to HDMLC plans. The difference between Halcyon V1 and V2 is only significant in CI of tumors less than 1cm in diameter. Halcyon plans required longer optimization than Truebeam VMAT plans, but had similar delivery efficiency.

**Conclusion:** For targets with diameter >1 cm, Halcyon's dual-layer stacked and staggered MLC is capable of producing similar dose conformity compared to HDMLC while reducing low dose spill to normal brain tissue. GI and V12Gy of Halcyon MLC plans were, in general, inferior to non-coplanar DCA or VMAT plans using HDMLC, likely due to coplanar geometry and wider MLC leaves. HDMLC maintained its advantage in CI for smaller targets with diameter <1 cm.

## Introduction

Stereotactic radiosurgery (SRS) has gained substantial popularity in the radiation oncology community, especially in smaller clinics. It has become a standard and effective way to manage brain metastases for cancer patients (1–4). There have been accumulated studies indicating that, for cancer patients with metastatic brain tumors, SRS provides effective and safe treatment compared to whole-brain radiation therapy (WBRT) alone, both as a monotherapy and combined with WBRT (5, 6). In addition, it has been reported that the deterioration of neurocognitive function following SRS is much less pronounced than WBRT, and therefore improves the quality of life for cancer survivors (5–7). Additional benefits of using SRS to control brain metastasis include substantial time and resource savings for the clinical care team, as well as preserving options of re-irradiation either to the remaining part of the brain or another part of the body.

The recent development of high-precision treatment delivery systems and image guidance expanded SRS treatment to linac-based radiation therapy centers. The use of volumetric-modulated arc therapy (VMAT) and high-definition multileaf collimators (HDMLC) enabled precise control of beam apertures to create conformal dose to the target while maintaining fast dose fall-off outside the target.

**Figure 1:**
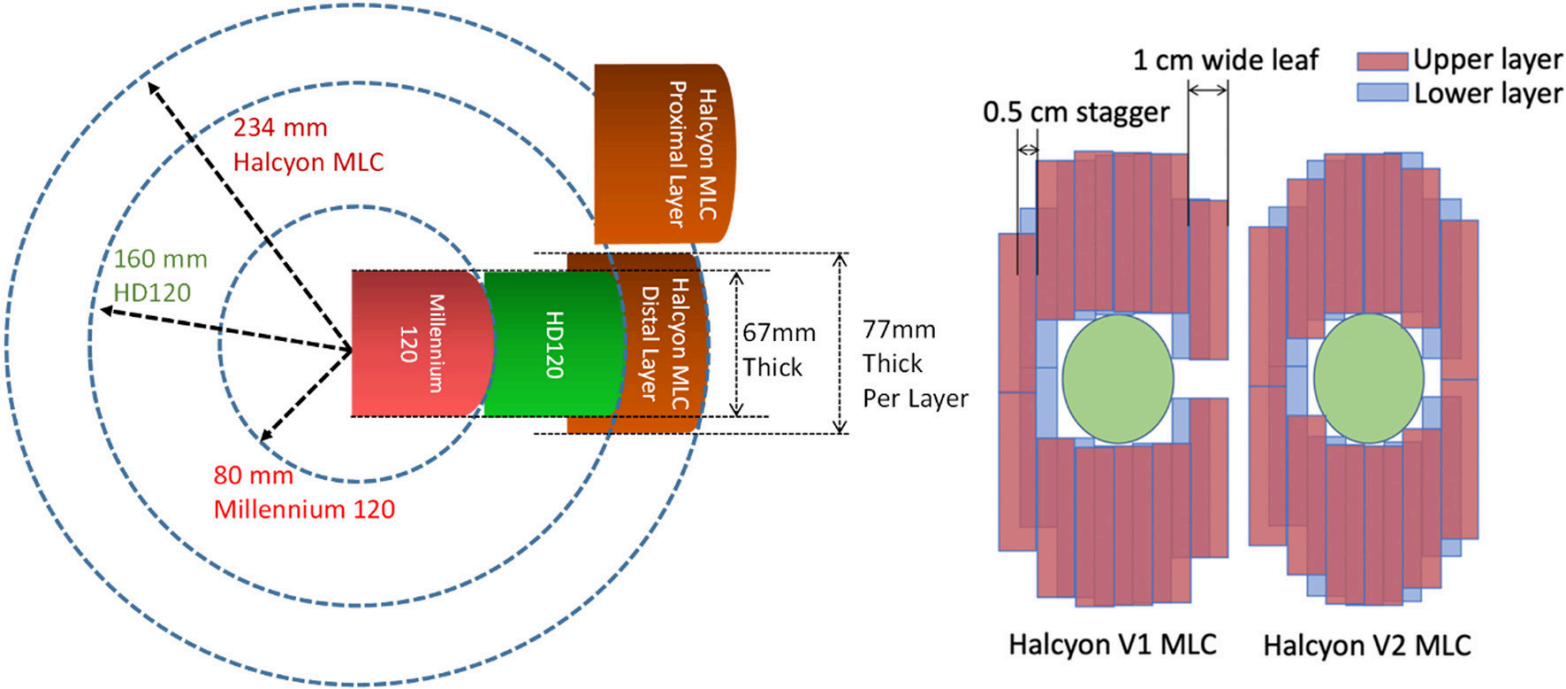
**(Left)** MLC Design comparison of current dual-layer Halcyon MLC (brown) vs. 0.25 cm wide HD-120 MLC (Green) and 0.5 cm wide Millenium-120 MLC (Red) showing increased leaf thickness and rounding radius. **(Right)** Different between Halcyon MLC version 1 and version 2. Halcyon V2 enables the upper layer to be used for beam shaping, effectively producing 0.5 cm modulation resolution.

More recently, a new type of straight-through linac has been introduced by Varian Medical Systems (Palo Alto, CA). The new linac, named Halycon™, offers improved mechanical design and treatment workflow. The gantry rotation is 4 times faster than a typical C-arm linac, such as the Truebeam® (Varian Medical Systems, Palo Alto, CA), with a ring-shaped enclosure which minimizes the possibility of collision. In addition, a new dual-layer stacked and staggered MLC design was introduced to this delivery system for a jaw-less configuration. This new type of MLC offers leaf speeds that are 2 times faster than traditional MLCs on Varian linacs, reduced leakage to ~0.05% (due to stacked layers and larger leaf height), and an improved penumbra with smaller dosimetric leaf gap (DLG) of 0.1 mm. This improved design, coupled with 6FFF energy, makes it a potentially favorable beam-shaping device for cranial treatment. Halcyon version 1 (V1) only uses lower MLC layer as beam-shaper and to modulate intensity/apertures while the upper layer MLC “ride along” the lower layer to provide additional shielding, similar to back-up jaws. So the modulation resolution of Halcyon V1 is limited at 1 cm perpendicular to the MLC traveling direction. Halcyon version 2 (V2) enables the upper layer to be used for beam shaping along with the lower layer, effectively producing 0.5 cm theoretical modulation resolution due to the 0.5 cm staggered design. Some physical design and functionality differences between Halcyon MLC and previous HD-120 MLC and Millenium-120 MLCs are illustrated in Figure 1, as well as the difference between Halcyon version 1 and version 2.

The current limitations of Halcyon MLC design compared to the Truebeam platform for the purpose of SRS are 2-fold. First, the MLC leaves are 1 cm wide with 0.5 cm offset between the upper and lower layers. This limits the theoretical modulation resolution to 0.5 cm when both layers are used. Compared to the widely accepted MLC design for SRS applications, which has 0.25 cm leaf width, the wider MLC leaves could lead to inferior dosimetric performance for small lesions. Second, the current Halcyon design does not allow couch rotation and, therefore, cannot generate non-coplanar beam arrangements that are typically used in SRS applications. This potentially has negative impact on dosimetry as well.

Nevertheless, Halcyon MLC exhibits lower leakage and a smaller penumbra, which may provide some benefits for SRS. Given the improvements and limitations of this new delivery system, it is important to understand how this system performs compared with other linac-based delivery configurations for multiple brain metastasis treatment. Due to the increasing popularity of performing SRS treatments at community centers, where the Halcyon platform was primarily designed for, we designed this comparative study. The study provides a benchmark dosimetry evaluation comparing Halcyon treatment plans to current standard treatment techniques for multiple brain metastasis with a single isocenter.

## Methods and Materials

**Figure 2:**
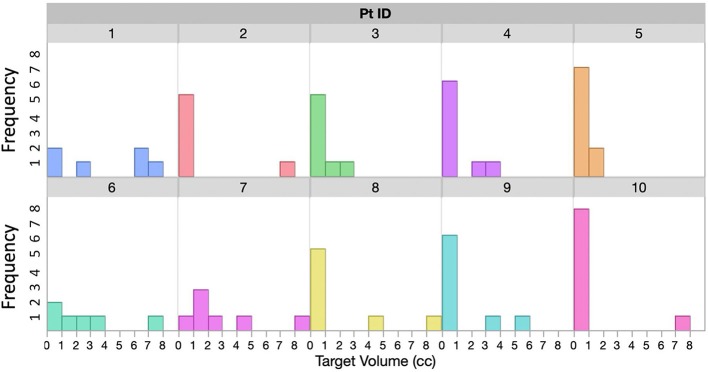
Distributions of tumor sizes for all 10 patients included in this study.

With IRB approval, 10 patients with multiple brain metastases treated with a single isocenter on a C-arm linac were retrospectively studied. The number of tumors treated for each patient in one session ranges from 6 to 10. Tumor size distribution for each patient is shown in Figure 2. For most patients there is at least one larger target receiving 15Gy−18Gy Rx, and several small targets receiving Rx between 21 and 24Gy, representing a wide distribution of target sizes and Rx levels that we commonly see in clinic.

**Figure 3:**
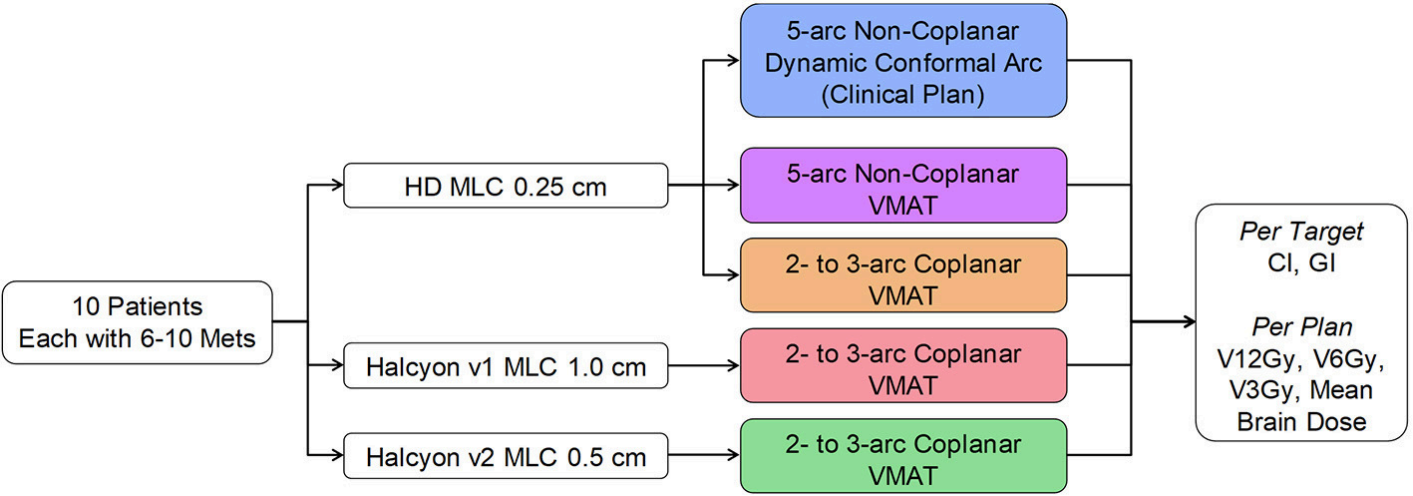
Study design flowchart.

Clinical treatment plans for these patients were developed using a single isocenter multi-aperture dynamic conformal arc technique implemented in the Brainlab Elements™ treatment planning system. Additional comparative treatment plans were generated in Eclipse™ treatment planning system using three different VMAT techniques: non-coplanar VMAT using 0.25 cm Truebeam HD-MLC, coplanar VMAT using 0.25 cm Truebeam HD-MLC, and Halcyon MLC. The reason that coplanar Truebeam HD-MLC was included here was not due to clinical reasons, but to provide a similar beam arrangement as in Halcyon. Figure 3 shows the study design diagram and different types of MLCs.

All treatment plans were developed with 6MV flattening-filter-free (6XFFF) energy and maximal dose rate for the type of linac: 1,400 MU/min for Truebeam, and 800 MU/min for Halcyon. Beam arrangement differs from technique to technique. For the original treatment plan developed using Brainlab Elements™ system, 5–9 arcs with 6MV flattened (6X) energy were used depending on the preconfigured templates. All arcs are dynamic conformal arcs in nature, i.e., the MLC only conforms to targets at all angles and are not used for modulation. All VMAT treatment plans followed one standard planning strategy reported by Clarks et al. (8), with the exception of beam arrangement. Isocenters were all placed automatically by the TPS at the center of mass of all targets combined. Non-coplanar VMAT plans used a 4-arc technique with 1 full (360°) arc at 0° couch rotation and 3 non-coplanar half (180°) arcs at couch rotation of 45°, 135°, and 270°. Coplanar VMAT plans used 2–3 arcs, depending on the number of targets, and maintained consistency across Truebeam and Halcyon plans. For all treatment plans, Photo Optimizer™ v15 was used for optimization and the Analytical Anisotropic Algorithm (AAA) v15 with calculation grid set to 1 mm was used for dose calculation. The optimization process is the same between Truebeam and Halcyon treatment plans. All plans were normalized so that the minimal coverage by the prescription dose to any targets is at or above 99% of target volume (V100% ≥ 99%).

Key dosimetric parameters were compared across treatment plans. The comparison was performed in two parts: per-target dosimetry comparison that included conformity index (CI) and gradient index (GI) for each target, and per-plan dosimetry evaluation on total brain V12Gy, V6Gy, V3Gy, and mean dose (D_mean_) to normal brain for the total treatment. V12Gy has been used as a key factor associated with radiation-induced necrosis (9, 10), and is therefore an important factor to assess. Per target paired comparisons across different planning and delivery platforms were conducted within two groups defined by equivalent target diameter >1 and ≤ 1cm. CI was calculated following the ICRU 62 definition (11): CI = V_Rx_/VPTV, where V_Rx_ is volume of the prescription dose and V_PTV_ is volume of the PTV. GI was calculated as defined by Paddick and Lippitz (12): GI = V_50%Rx_/V_100%Rx_, where V_50%Rx_ is the volume of the 50% isodose line and V_100%Rx_ is the volume of the 100% isodose line. Since all targets were normalized to have prescription dose covering at least 99% of the target volume, target coverage is already ensured. Therefore, ICRU CI was used to highlight excessive dose outside the target following Clarks et al. In plans where a different prescription was used for different targets based on the size, GI was calculated based on the corresponding prescription dose to each particular target. Homogeneity index (HI), defined as Dmax/Rx, was documented but not included in paired comparison. This is because maximal dose (Dmax) was not constrained during optimization following guidelines by Clarks et al. and our institutional practice, therefore HI does not directly reflect each system's ability to achieve planning objectives. Wilcoxon signed rank test (13–15) was used as a non-parametric test for paired data based on differences between paired dosimetric parameters of plans generated by different planning and delivery platforms. The test is based on ranks of the difference values between paired samples, and, unlike *t*-test, does not assume any distribution characteristics of the underlying population. Statistical significance level was set at *p* < 0.05.

To assess optimization and delivery efficiency across multiple planning techniques, optimization time was recorded for 5 patients, and delivery efficiency were assessed for all 10 patients by comparing total MU and estimated delivery time. Because DCA plans do not involve VMAT optimization, it was excluded from the optimization efficiency comparison. Estimated delivery time was calculated by considering the actual machine limits (dose rate, gantry rotation, MLC speed). Because of the high MU nature of all SRS plans (>3,000 MU per arc), the limiting factor for delivery speed is found to be machine dose rate for all cases. Therefore, estimated delivery time was calculated from total MU by using machine-specific maximal dose rate of 1,400 MU/min for HDMLC plans, 800 MU/min for Halcyon plans, and 600 MU/min for DCA plans, plus 1 min per non-zero couch rotation angles to allow for setup and verification process for each non-coplanar arc.

## Results

### Per Target Dosimetry Analysis

**Figure 4:**
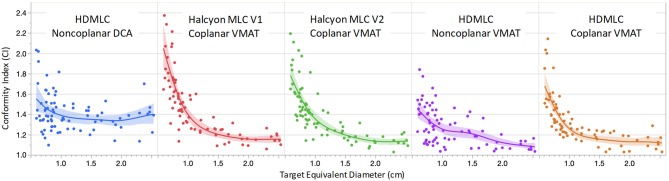
Comparison of conformity index (CI) as a function of target equivalent diameters for five planning and delivery techniques. Dots are the actual CI for an individual target. Solid lines are fitting lines using spline model and the shaded areas are 95% confidence interval of fit.

Figure 4 shows the CI comparison across multiple planning and delivery techniques, fitted to the equivalent target diameter calculated by treating the targets as spherical. According to radiosurgery quality assurance guidelines established by RTOG, a CI above 2.0 is considered as a minor deviation from the guideline and a CI of 2.5 and above is considered as a major deviation from the guideline (16). From the data shown in Figure 4, all of the targets had CIs < 2.5 with the majority of the targets' CI below 2.0. This indicates that, from the conformity point of view, all techniques and MLC designs satisfied the clinically acceptable requirement.

For targets < 1 cm in diameter, non-coplanar plans in general achieved better conformity compared to coplanar plans. Within coplanar plans, Halcyon V2 MLC showed improved CIs for small targets compared to V1 MLC. V2 MLC also performed very similarly compared to CIs achieved with HD MLC, which had twice finer modulation resolution perpendicular to leaf travel (0.25 cm for HD MLC vs. 0.5 cm for Halcyon V2 MLC). For targets with diameter larger than 1 cm, all VMAT plans, including Halcyon V1 MLC and V2 MLC plans, exhibited superior CI compared to clinical DCA plans. The CI performance difference among all VMAT plans are not significantly different for targets larger than 1 cm.

**Figure 5:**
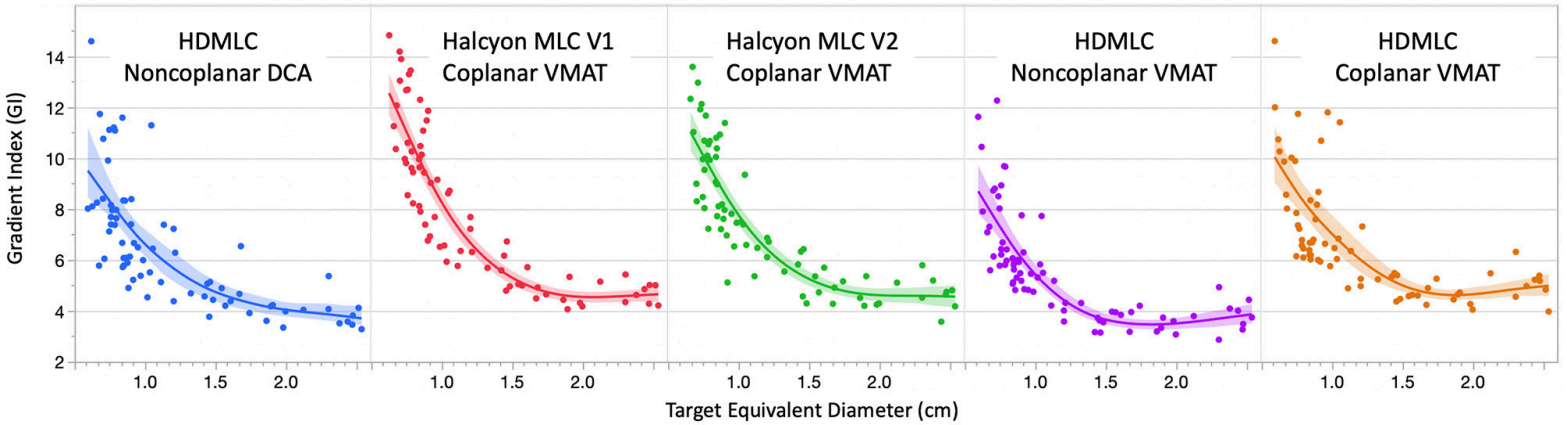
Comparison of gradient index (GI) variation as a function of target equivalent diameters for five planning and delivery techniques. Dots are the actual GI for an individual target. Solid lines are fitting lines using spline model and shaded areas are 95% confidence interval of fit. Only GIs < 15 are included in the graph to avoid data skewing due to bridging 50% isodose lines.

Figure 5 compares GI as a function of target equivalent diameter across 5 delivery and planning techniques. Seven targets (35 data points), out of 80, were not included in the GI fitting graph due to the bridging of their 50% isodose volume with another nearby target. When a target's 50% isodose volume bridges with another target's, the V50% no longer describes dose fall off around one single target, and therefore cannot characterize the MLC system. However, this does not mean that the overlap region is neglected. In fact, the overlap region and its dosimetric impact is fully evaluated in the V12Gy analysis later. Overall, non-coplanar techniques achieved lower GI compared to coplanar solutions. Comparison across coplanar plans suggested that Halcyon MLC in general had inferior GI for targets smaller than 1 cm in diameter, but comparable GI for targets larger than 1 cm in diameter when compared to HD MLC.

**Figure 6:**
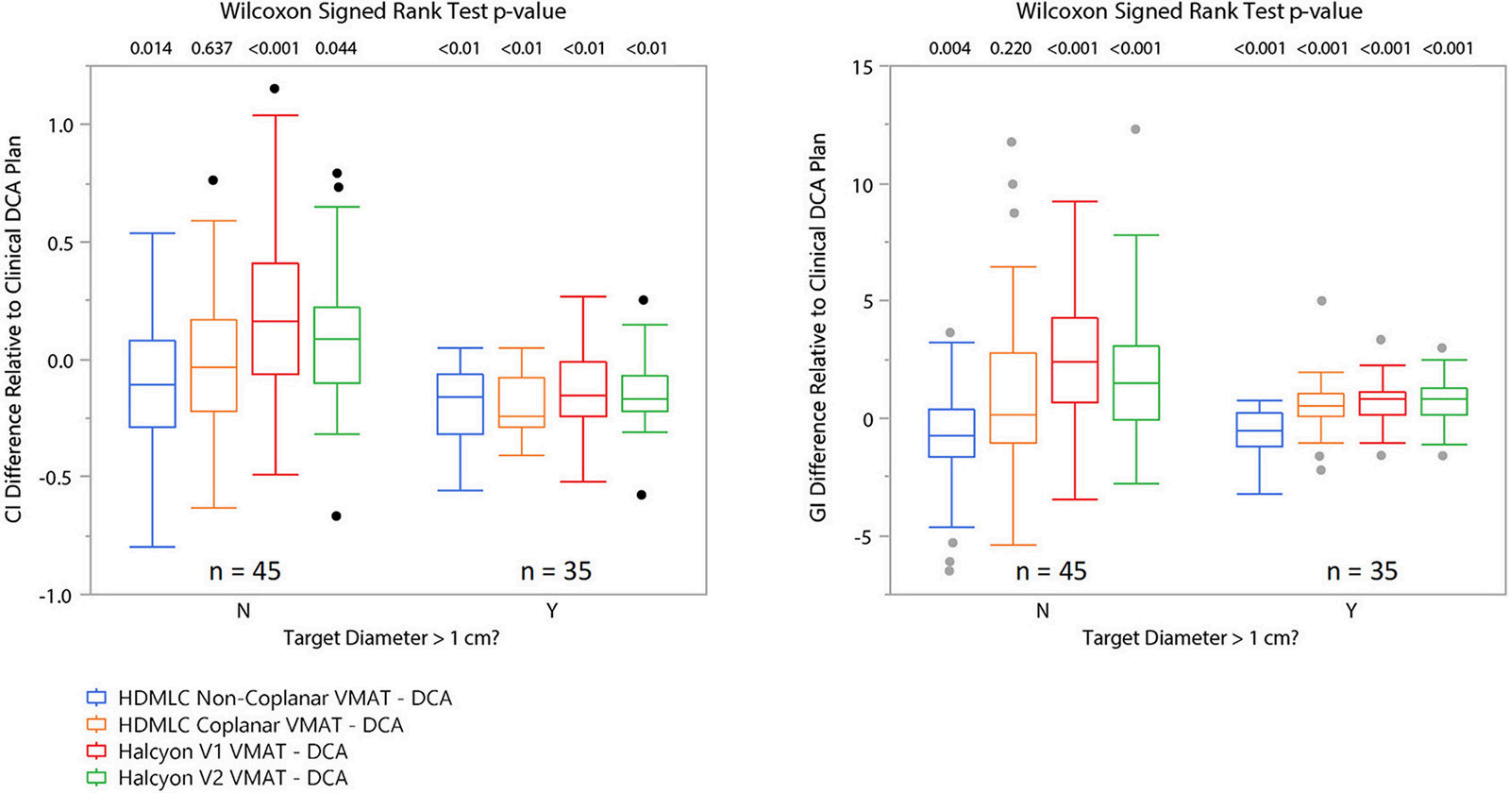
Matched pair comparison of CI and GI between each VMAT technique and the corresponding clinical DCA plans separating cases into two groups by equivalent target diameter. N means target diameter is ≤ 1 cm and Y means target diameter is >1 cm. Wilcoxon signed rank test was used to assess statistical significance of the difference between VMAT plan's CI/GI and those of the clinical DCA plans.

Figure 6 visualizes a paired CI and GI comparison with respect to clinical baseline plan. Since we used clinical non-coplanar DCA plans with HDMLC as baseline, in this comparison all VMAT plans' CI and GI are presented relative to the DCA plans' CI and GI in terms of absolute difference in value; i.e., *CI*_*VMAT*_−*CI*_*DCA*_ and *GI*_*VMAT*_−*GI*_*DCA*_. Based on observations in Figures 4, **5**, the boxplots are also divided into two sections for targets with diameter larger (35 targets) or smaller (45 targets) than 1 cm.

The CI and GI differences between techniques for each target are also analyzed and summarized in Table 1, with the targets separated based on their diameter (>1 or < 1cm). For each case, the CI or GI for the corresponding technique located in the first column of each table is subtracted from the CI or GI for the corresponding technique located in the top row, respectively. The mean, standard deviation, and Wilcoxon Signed Rank test results of all per-case CI or GI differences are then summarized and shown in the table cell. Positive mean values in the table cells indicate that the corresponding technique in the top row produced on average a higher CI or GI compared to the corresponding technique in the first column on the left; and negative values indicate the opposite. Wilcoxon Signed Rank test was used to determine the statistical significance in the difference and the *p*-values are reported alongside the difference. Values that are statistically significant (*p* < 0.05) are bolded for clarity.

**Table 1:** Mean and standard deviation values of per-target paired CI/GI difference between different planning techniques.

	**HD MLC non-coplanar VMAT**	**HD MLC coplanar VMAT**	**Halcyon MLC V1 coplanar VMAT**	**Halcyon MLC V2 coplanar VMAT**
**(A) CI difference (top - left) for targets with diameter < 1 cm**				
HD MLC non-coplanar DCA	**−0.112 ± 0.276 (*****p*** **= 0.014)**	–0.016 ± 0.294 (*p* = 0.637)	**0.226 ± 0.363 (*****p*** **< 0.001)**	**0.089 ± 0.284 (*****p*** **= 0.044)**
HD MLC non-coplanar VMAT		0.096 ± 0.260 (*p* = 0.065)	**0.338 ± 0.381 (*****p*** **< 0.001)**	**0.200 ± 0.300 (*****p*** **< 0.001)**
HD MLC coplanar VMAT			**0.242 ± 0.241 (*****p*** **< 0.001)**	**0.105 ± 0.185 (*****p*** **< 0.001)**
Halcyon MLC V1 coplanar VMAT				**−0.137 ± 0.232 (*****p*** **< 0.001)**
**(B) CI difference (top - left) for targets with diameter > 1 cm**				
HD MLC non-coplanar DCA	**−0.194 ± 0.149 (*****p*** **< 0.001)**	**-0.193 ± 0.13 (*****p*** **< 0.001)**	**−0.129 ± 0.164 (*****p*** **< 0.001)**	**−0.154 ± 0.148 (*****p*** **< 0.001)**
HD MLC non-coplanar VMAT		0.001 ± 0.135 (*p* = 0.596)	**0.065 ± 0.149 (*****p*** **= 0.002)**	0.040 ± 0.135 (*p* = 0.084)
HD MLC coplanar VMAT			**0.064 ± 0.095 (*****p*** **< 0.001)**	**0.039 ± 0.093 (*****p*** **= 0.016)**
Halcyon MLC V1 coplanar VMAT				–0.025 ± 0.074 (*p* = 0.082)
**(C) GI difference (top - left) for targets with diameter < 1 cm**				
HD MLC non-coplanar DCA	**−1.12 ± 2.80 (*****p*** **= 0.004)**	0.34 ± 4.71 (*p* = 0.220)	**1.76 ± 4.34 (*****p*** **< 0.001)**	**1.52 ± 3.66 (*****p*** **< 0.001)**
HD MLC non-coplanar VMAT		**1.46 ± 3.53 (*****p*** **< 0.001)**	**2.88 ± 3.23 (*****p*** **< 0.001)**	**2.641 ± 2.66 (*****p*** **= 0.001)**
HD MLC coplanar VMAT			**1.41 ± 3.03 (*****p*** **= 0.002)**	**1.18 ± 4.06 (*****p*** **= 0.020)**
Halcyon MLC V1 coplanar VMAT				−0.24 ± 2.543 (*p* = 0.136)
**(D) GI difference (top - left) for targets with diameter > 1 cm**				
HD MLC non-coplanar DCA	**−0.66 ± 0.95 (*****p*** **< 0.001)**	**0.62 ± 1.17 (*****p*** **< 0.001)**	**0.73 ± 0.8896 (*****p*** **< 0.001)**	**0.66 ± 0.9276 (*****p*** **< 0.001)**
HD MLC non-coplanar VMAT		**1.28 ± 1.146 (*****p*** **< 0.001)**	**1.39 ± 1.007 (*****p*** **< 0.001)**	**1.32 ± 0.8818 (*****p*** **< 0.001)**
HD MLC coplanar VMAT			0.11 ± 0.906 (*p* = 0.350)	0.04 ± 1.075 (*p* = 0.200)
Halcyon MLC V1 coplanar VMAT				–0.06 ± 0.623 (*p* = 0.810)
**Bold** indicates statistical significance based on Wilcoxon Signed-Rank Test				

*Values shown were generated using CI and GI from the corresponding technique indicated in the top row minus the technique indicated in the left-most column. Positive mean values in the table cells indicate that the corresponding technique in the top row produced on average a higher CI or GI compared to the corresponding technique in the first column on the left; and negative values indicate the opposite. Bold text indicates statistical significance found between the corresponding techniques using Wilcoxon Signed Rank test*.

Overall, non-coplanar VMAT plans exhibited superior CI and GI, which can be seen in Figures 4–6, as well as in Table 1 where all mean CI/GI differences relative to non-coplanar VMAT plan are positive (3rd row in each sub-table). Regardless of tumor size, all coplanar techniques (both Halcyon MLC and HDMLC) had inferior GI compared to DCA plans (positive mean values in 2nd row, column 3–5 of Sub-Tables 1C,D). For small targets with diameter < 1cm, Halcyon V1 and V2 plans exhibited statistically significant increase in CI and GI (last two columns in Sub-Tables 1A,C) compared to all HDMLC plans including DCA and VMAT. Compared to Halcyon V1, Halcyon V2 improved CI for small targets but not GI (lower-right cell in Sub-Tables 1A,B). For targets with diameter > 1 cm, all VMAT plans, including Halcyon V1 and V2, achieved significantly better CI than DCA plans as shown by negative mean values in the 2nd row of Sub-Table 1B. Halcyon V2 MLC using coplanar beams achieved similar CI compared to non-coplanar VMAT with HDMLC but inferior GI (3rd row last column in Sub-Table 1B,D). The differences in GI for large tumors between all coplanar techniques, including Halcyon V1, V2, and HDMLC in coplanar setting, were not significant (row 4–5 column 4–5 in Sub-Table 1D).

The mean homogeneity index (HI) for each technique (with 95% confidence interval) is as follows: 1.26 (1.23–1.30) for non-coplanar DCA, 1.41 (1.38–1.44) for Halcyon V1, 1.42 (1.38–1.45) for Halcyon V2, 1.40 (1.37–1.43) for non-coplanar VMAT with HDMLC, and 1.53 (1.49, 1.56) for coplanar VMAT with HDMLC. These results are consistent with findings published by other groups (8) and are well below the published HI of GammaKnife-based SRS (1). It should be noted that these HI statistics were based on SRS plans optimized without maximal dose objectives and could substantially change if maximal dose objectives were used during optimization.

### Per Patient Dosimetry Analysis

On a per-patient level, clinicians care about the total excessive dose delivered to the normal brain tissue, as well as key organs at risks. Therefore, in the following section, the normal tissue dose matrices were examined.

**Figure 7:**
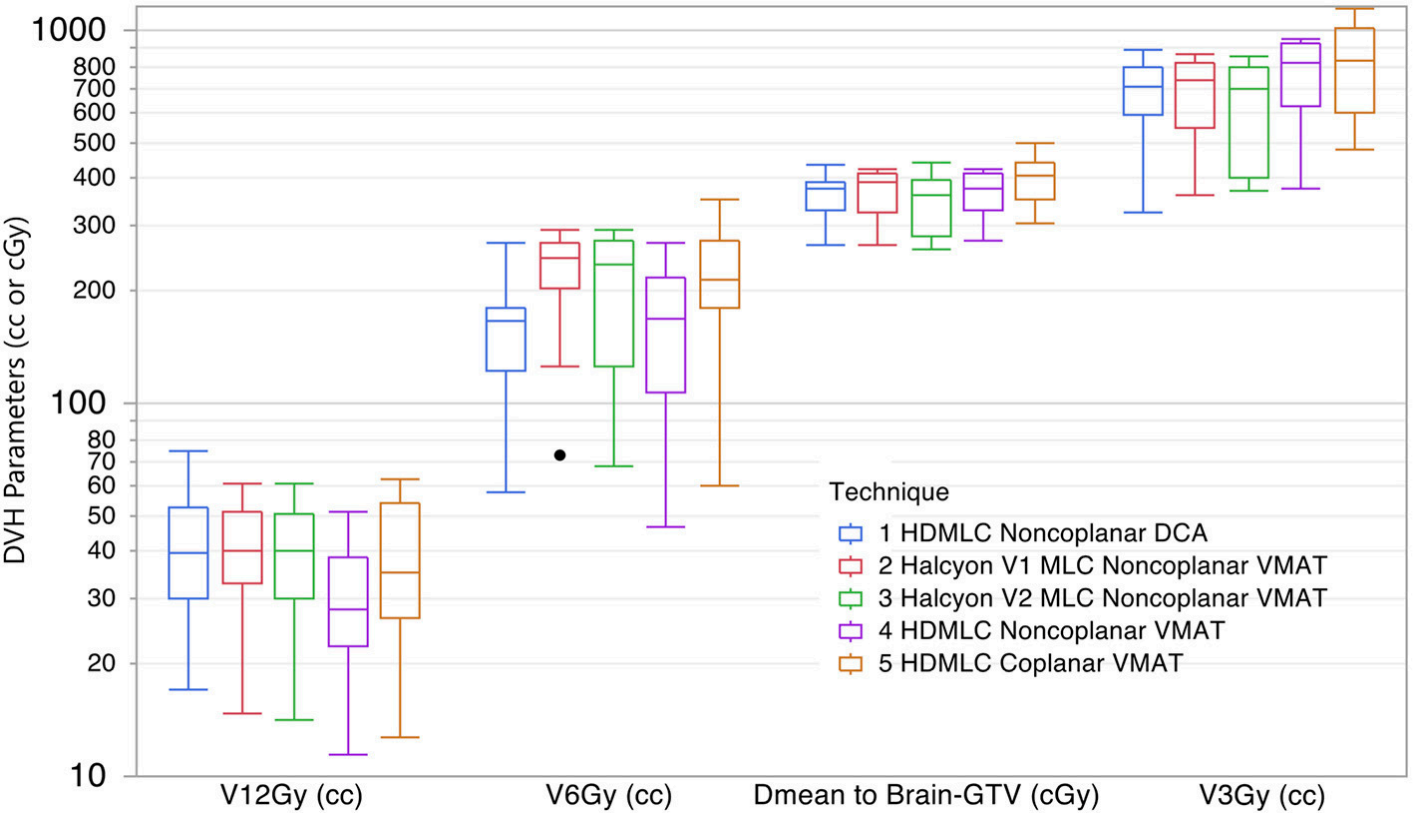
Comparison of dose spillage to normal brain tissue from 5 different techniques. Parameters shown are V12Gy, V6Gy, and V3Gy in cc, and mean dose to brain-GTV volume in cGy. Within each box there are 10 plans summarized.

The dose spillage to normal brain tissue at multiple dose levels are compared in Figure 7. To better visualize a wide range of volume parameters, the vertical axis is displayed in log scale. V12Gy is of particular interest because of its correlation to radionecrosis following SRS treatment. Non-coplanar VMAT with HD MLC outperformed all other techniques with a significantly lower V12Gy (*p* = 0.002 for paired comparison with all other groups). However, both Halcyon MLC plans performed very similar to non-coplanar DCA plans, and slightly better than coplanar HD MLC plans. For V6Gy, both non-coplanar techniques outperformed coplanar techniques; however, V6Gy between Halcyon and HD MLCs were very similar. Mean dose to Brain-GTV was found to be similar for all techniques regardless of arc arrangement or MLC. As an indicator of low dose spillage to brain tissue, V3Gy comparison showed that Halcyon V2 offered improved low dose volume to normal brain tissue compared to both coplanar (*p* = 0.004) and non-coplanar (*p* = 0.01) plans using HD MLC. Difference in V3Gy between DCA and Halcyon V2 VMAT plans was not significant (*p* = 0.557). These results demonstrated the advantage of the low leakage MLC design in Halcyon.

**Figure 8:**
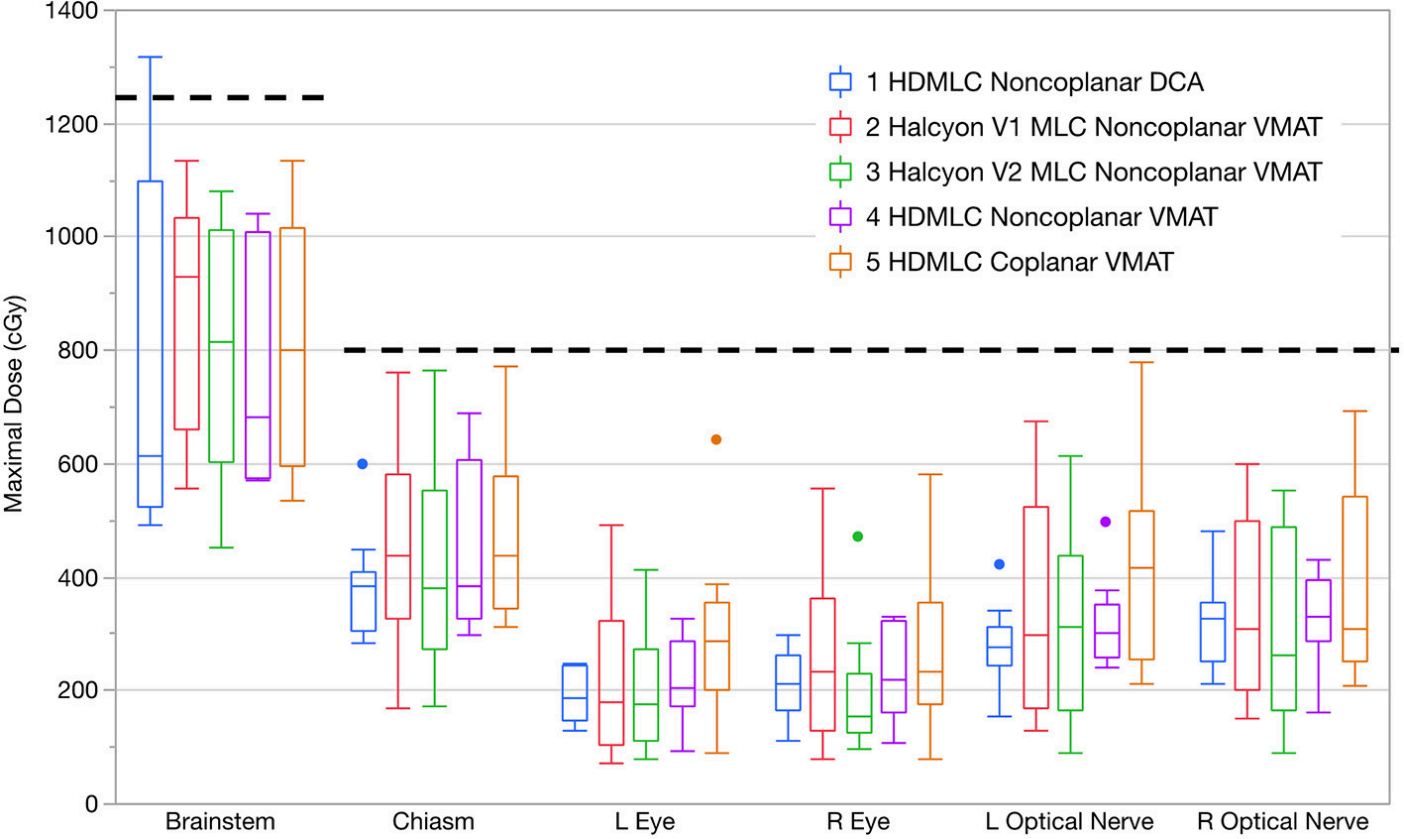
Maximal doses to key OARs compared in boxplots across all planning and delivery techniques. Thick dashed lines show the clinical constraints used. Within each box there are 10 plans summarized. Dots indicate outliers defined by 1.5 interquartile range (IQR).

In addition to normal brain tissue, doses to several critical organs-at-risk were also assessed according to their dose constraints. The results are shown in Figure 8. All VMAT plans met clinical constraints on OARs. No substantial differences were observed across different MLC and planning techniques.

### Optimization and Delivery Efficiency

Results for optimization and delivery efficiency were recorded for five patients under the same calculation configuration. Optimization times were similar across different treatment planning strategies within both the Truebeam and Halcyon platforms, but differed substantially between the two. Truebeam optimization times ranged from 15 to 24 min while Halcyon optimization times ranged from 28 to 40 min.

**Figure 9:**
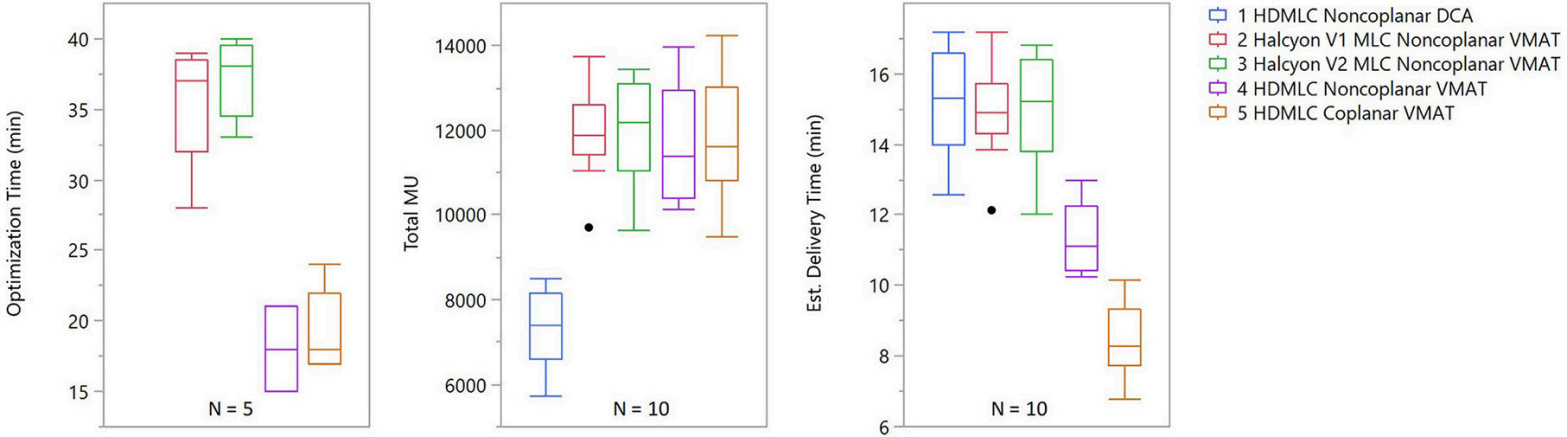
Optimization and delivery efficiency comparison across different planning strategies. For optimization, only 5 patients were included and, because it does not include VMAT optimization, DCA was omitted from the optimization time chart. For delivery efficiency, both total MU and estimated delivery time are shown. Estimated delivery time was calculated using the dose rate and gantry rotation speed limits plus 1 min per non-zero couch angle due to additional time required for setup and verification.

Delivery efficiency was assessed by total MU and estimated delivery times of treatment plans were generated for the different planning strategies. From Figure 9 it can be seen that the total MU were similar across all VMAT techniques, regardless of machine type or arc arrangement. No statistical significance was found in any paired tests between VMAT techniques. DCA plans had substantially lower MU than VMAT plans, which is expected due to the absence of modulation. The estimated delivery time showed that Halcyon plans in general had higher delivery times than HDMLC plans. This is mainly due to the maximal dose rate limit of 800 MU/min for the current Halcyon platform, compared to 1,400 MU/min for HDMLC with 6FFF energy on the Truebeam platform.

## Discussions

In this study, the performance of the Halcyon dual layer stacked and staggered MLC on small target dosimetry was quantified in the context of single isocenter multi-target radiosurgery. The purpose of this study was to explore the strengths and weaknesses of this new MLC design when it comes to treating multiple isolated small targets. We identified that size of the tumor (>1 or < 1 cm) played an important role in the performance comparison study. When considering treating very small cranial lesion, < 1 cm, preference would be to treat them in a clinic equipped with HD MLCs and non-coplanar delivery capabilities.

The results also demonstrated that Halcyon's 1 cm-wide MLC, with its staggered design, is capable of producing a conformity index similar to that achieved by a much finer MLC (0.25 cm width) for tumors >1 cm in diameter. It is worth noting that, for these tumors, the Halcyon actually achieved better CI compared to clinical plans generated using the DCA technique. For smaller tumors with diameter < 1 cm, the CIs achieved by using Halcyon MLC were found to be inferior when compared to using HD MLCs, but still met clinical constraints for the majority of cases. GI analysis showed the Halcyon MLC was capable of achieving similar GI compared to HD MLC in a coplanar setting for tumors larger than 1 cm in diameter, but slightly inferior for smaller tumors.

In both Figures 4, 5 there were targets outside 95% confidence-of-fit region (color-shaded area). It should be noted that this band only represents the uncertainty in the estimate of the fitted line at different target diameters, and is not a direct indication of the data distribution. One possible explanation for the spread of these outliers is the low dose interaction between different targets. If only one target is treated, the CI/GI should mostly be determined by MLC and planning techniques. However, when there are multiple targets being treated at the same time, dose fall-off region between nearby targets could overlap, either due to limited blocking resolution of the MLC or the proximity of the two targets. This could cause CI/GI being affected by parameters other than the diameter of the target. The smaller the target, the more likely this dose spillage could impact its CI/GI calculation, therefore higher spread is seen in general for smaller targets.

It should be noted that these results were based on using the same optimization constraints across all platforms to standardize the experiment condition and highlight potential differences due to MLC and arc arrangement limitations. It is possible to modify objectives for Halcyon platform to further improve some dosimetric parameters while maintaining target coverage and OAR doses. However, more stringent objectives are likely to increase the level of modulation and, therefore, the total MU. Multi-criteria optimization could be used to explore optimal trade-off between target coverage, dose fall-off, OAR doses, and delivery efficiencies.

One of the major limitations of the current Halcyon platform design is the omission of a rotatable couch, i.e., without yaw correction. This limitation could be the main reason for Halcyon's inferior GIs compared to clinical plans and non-coplanar VMAT, because coplanar VMAT, even with finer HD MLC, exhibited similarly inferior GIs. In addition, the MLC leaf width could also be a contributor to the difference observed on GI, as suggested by previous publications comparing leaves with different width on SRS dosimetry (17). Detailed small field profile analysis would also be helpful in understanding the Halcyon MLC's performance in terms of beam shaping and penumbra region characteristics.

**Figure 10:**
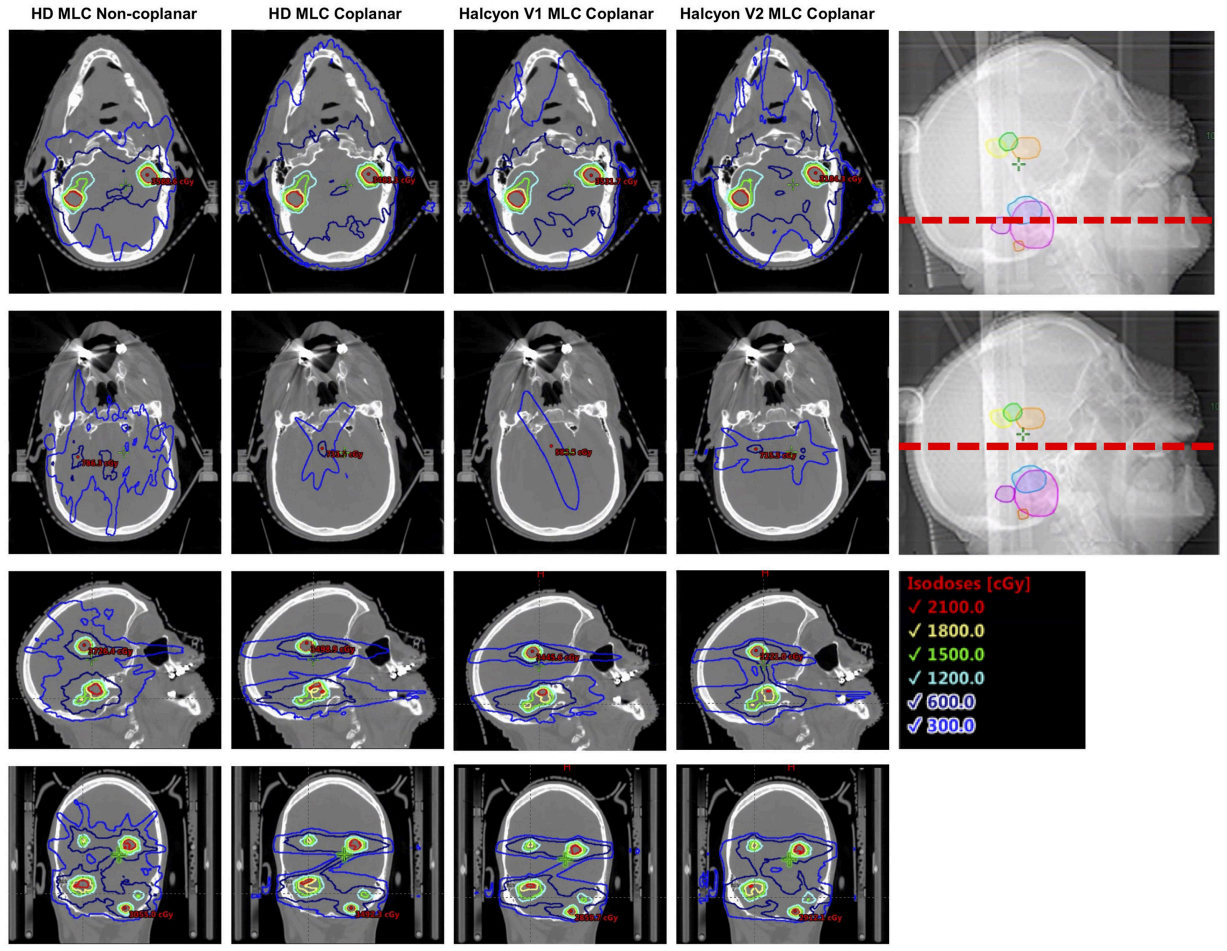
Illustration of dose fall-off characteristics across multiple different delivery techniques, both in and outside the target plane. Non-coplanar beam arrangements in general have better dose fall-off in the target plane (top row), but bears more low dose spread to tissue between target planes (2nd row).

However, in a clinical setting, especially for multiple targets scenario, total V12Gy should also be considered in addition to individual GI when assessing dose fall-off (18). It has been shown in the results section that, even with the limitation of coplanar beam arrangement, Halcyon achieved similar total V12Gy per plan as the clinical DCA plan. Figure 10 illustrates a representative case where non-coplanar beam arrangements exhibited better dose fall-off within the target plane in the axial direction, but increased low dose spread to tissue located between target planes. This, in part, reduced the advantage of having non-coplanar beams, and could explain why non-coplanar DCA plans had comparable V12Gy to coplanar arrangements.

Another benefit using Halcyon MLCs was the reduced volume of normal brain receiving low dose (3Gy) as well as lower mean dose to the normal brain tissue, as shown in Figure 7. This is likely due to the improved leakage characteristics of the dual-layer MLC design (< 0.1%) compared to traditional MLC design (~1%). For SRS treatment, very high cumulative monitor units (6,000-10,000) are often delivered. This is especially true for multi-target delivery, where the jaw has to be open to encompass multiple targets while the majority of the jaw opening is blocked by the MLCs. This makes the MLC transmission the dominate factor on effecting normal tissue dose. Reduction from 1 to 0.1% for a 10,000 MU delivery translates to a reduction from 100 to 10cGy in reference calibration condition. This reduction in normal brain dose could be meaningful for re-irradiation, as patients sometimes go through multiple sessions of SRS to manage their brain metastases. For all HDMLC plans in this study the jaw-tracking technique was enabled during optimization. It has been shown that enabling jaw tracking, in part, helps with reducing low dose spillage to the normal brain tissues (19). If the jaw tracking technique is not available, it is expected that the normal brain tissue receiving low dose (e.g., V3Gy) will further increase for HDMLC plans.

It should be emphasized again that this is strictly a planning study to compare the achievable SRS plan quality that this newly introduced MLC design is capable of, and does not fully warrant the use of Halcyon for SRS treatment even if the dosimetry is acceptable. Other factors, such as delivery accuracy as a result of small fields and the imaging system's coincidence with the treatment beam system, need to be well characterized before implementing this technique on Halcyon. To ensure accurate treatment delivery of planned dose, long term mechanical stability of the Halcyon system will also need to be well characterized. Such characterization should include Winston-Lutz or similar tests, MLC positioning accuracy as a function of gantry angle for both layers, and IGRT system performance. In addition, the omission of the ability to perform 6D couch correction will require a 6D adjustable mask or frame system to be used to perform rotational corrections around pitch, roll, and yaw axes. This is particularly important for multi-target treatment, as targets away from isocenter are very sensitive to residual rotational mismatches between the planned and treatment positions.

Nevertheless, the data presented in this study show the great potential of this dual-layer stacked and staggered MLC design, despite the current limitation on non-coplanar delivery. The conformity performance for targets > 1cm, coupled with reduced leakage, are likely to translate into additional benefit to other disease site applications where non-coplanar beam arrangements are not typically used.

## Conclusion

A new dual-layer stacked and staggered MLC design implemented in the Halcyon treatment delivery system has been evaluated for the performance on single-isocenter multi-target cranial treatment. Compared to clinical non-coplanar dynamic conformal arc (DCA) plans, the system was found to have comparable CI for targets >1 cm in diameter but inferior GI, likely due to the limitation that only coplanar beam arrangements can be used. V12Gy generated by Halcyon in a coplanar setting was found to be similar to clinical non-coplanar DCA plans. Reduced low dose spillage (V3Gy) was observed for Halcyon plans compared to Truebeam plans, likely as a result of reduced leakage in the new dual-layer MLC. Overall, the new MLC showed great potential in conforming to a small target while improving normal tissue blocking during high MU treatment, but its advantage is limited due to the current machine design which does not allow non-coplanar arcs. Planning quality using the current Halcyon™ design is clinically acceptable for treatments of multiple targets with diameter >1 cm. However, for targets with diameter < 1 cm, a unit with HDMLC and ability to treat with non-coplanar fields appears superior to the current Halcyon design. Future hardware and software developments are needed to extend the capability of Halcyon to treat lesions smaller than 1 cm.

## Ethics Statement

This study was performed under the IRB approval from Thomas Jefferson University.

## Author Contributions

TL designed the study, performed experiments and data analysis, and drafted the manuscript. PI assisted with data collection and manuscript drafting. HL, WS, MA-B, WZ, B-KT, JM all contributed substantially to the concept and design of the study. LD oversaw the study and manuscript preparation.

### Conflict of Interest Statement

This work is partially supported by Varian Medical Systems. MA-B received honorarium from Varian Medical Systems for work outside submitted work. JM is on the advisory board of and obtained grant funding unrelated to this work from Varian Medical Systems. LD received grant funding unrelated to this work from Varian Medical Systems. The remaining authors declare that the research was conducted in the absence of any commercial or financial relationships that could be construed as a potential conflict of interest.
